# Changing pattern of infective endocarditis in Iran: A 16 years survey

**DOI:** 10.12669/pjms.291.2682

**Published:** 2013

**Authors:** Behnam Hajihossainlou, Mohammad-Ali Heidarnia, Babak Sharif Kashani

**Affiliations:** 1 Behnam Hajihossainlou, MD, Graduate Student in Clinical Research Program, Shahid Beheshti University of Medical Sciences, Tehran, Iran.; 2 Mohammad-Ali Heidarnia, MD, Assistant Professor, Department of Epidemiology, Shahid Beheshti University of Medical Sciences, Tehran, Iran.; 3 Dr. Babak Sharif Kashani, MD, Assistant Professor, Department of Cardiology, Shahid Beheshti University of Medical Sciences, Tehran, Iran.

**Keywords:** Infective Endocarditis, IDUs, Vegetation size

## Abstract

***Objectives:*** To investigate the changes in characteristics of patients with infective endocarditis in Iran and comparing the results with the changing profiles of Infection Endocarditis (IE) in other countries.

***Methodology:*** We studied all patients with definite or possible IE seen at four referral teaching hospitals in Iran from Jan. 1995 to Dec. 2010. The data was analyzed both collectively and separately in two consecutive eight-year periods, i.e. 1995-2003 and 2004-2010.

***Results:*** A total of 286 episodes of IE, 172 males and 114 females, were reviewed from which 162 ones were in the first eight-year time period and 124 episodes in the second one. Mean age of the patients was significantly increased in the second eight-year period (24.2±11 vs 39.4±15 years old, p value = 0.01). Increase in the episodes caused by Staphylococcus aureus was significant (40.7% vs 22.8%, p value = 0.01). The mean size of the vegetation was noticeably higher among IDUs than non-IDUs (1.53±0.1cm vs 0.76±0.2cm, p value < 0.001). As well as extra cardiac complications, mortality rate was noticeably higher among the patients with vegetation size ≥ 1cm (34.4% vs 16.3%, p value = 0.003). There was not a significant difference regarding the mortality rate between the conservatively and surgically treated patients (20.7% vs 22.9%, p value = 0.07).

***Conclusion:*** The most important changing characteristic of IE which influences the outcome of the disease seems to be vegetation size which can account for as the outcome predictor.

## Introduction

 Known as a serious infection of heart valves, Infective Endocarditis (IE) is still associated with high mortality and morbidity despite advances in medical and surgical interventions.^1^^-^^3^ Historically, infective endocarditis was a disease which occurred predominantly in patients with an underlying heart problem, particularly, congenital heart disease (CHD) and Rheumatic heart disease (RHD). It was mostly a result of community acquired bacteremia including infections acquired following invasive dental or medical procedures or during the hospitalization in medical centers. Streptococcus viridans was the most common causative agent and compared to other heart valves, aortic and mitral valves were more frequently involved.^2^^-^^5^

 Over the past two decades, new trends in the epidemiology of IE have been noticed which influenced the manifestations and outcome of the disease.^4^^-^^8^ Recent studies indicate a significant change in the population at risk, causative microorganisms, involved valves and survival of the patients.^7^^-^^13^ For instance, the prevalence of RHD is decreasing while the number of clinically ill patients with IE is increasing.^9^^,^^10^ Endocarditis has increasingly become a disease of the elderly.^11^ In the developed countries, about one-half of all IE cases occurs in patients over the age of 60 and the median age of patients has increased steadily during the past decades.^3^^,^^12^

 There is limited information about the characteristics of IE and its potential changes in Iran compared to the past. In this study, we investigated the situation of all patients with definite or possible IE seen at four referral teaching Medical Centers affiliated by Shahid Beheshti University of Medical Sciences (Loghman Hakim, Shahid Modarres, Labbafinejad and Bu-Ali Hospitals) in Tehran, Iran, from Jan. 1995 to Dec. 2010 in an effort to identify the changes in the characteristics of patients with infective endocarditis in Iran and to compare the results with the changing profile of IE in other countries.

## Methodology

 We reviewed medical records of all patients with a discharge diagnosis or postmortem findings of IE hospitalized in the four teaching hospitals from January 1995 to December 2010. These hospitals are considered as tertiary referral hospitals for infectious, cardiac and cardiothoracic surgical diseases, serving patients from all over the country.

In this study, the cases of IE which didn’t meet the Duke criteria for definite or possible IE were excluded. We also excluded the patients’ records which didn’t contain expected details. In the case of microbiologic findings, we considered three sets of blood cultures drawn one hour apart before the introduction of antibiotic treatment as required including factor. For echocardiographic studies, at least, one transthorasic echocardiography which contained enough details, particularly about vegetation size, was demanded. Using medical records, we also extracted required epidemiologic and demographic data including age, sex, predisposing conditions such as heart diseases, recent surgical procedures, intravenous drug injection, underlying diseases and co-infections.

 In order to define the potential changes in the clinical presentation of the patients with IE, this study was divided into two chronologic parts; the first eight-year period (from January 1995 to December 2002) and the second eight-year period (from January 2003 to December 2010). The characteristics of the disease were analyzed both collectively (in whole 16 years) and separately (in eight-year intervals).

 Chi-square test was used to analyze the differences between the eight-year time periods in distributions of the categorical variables. Student “t” test and one-way analysis of variance were done to analyze the mean differences. In order to compare the blood culture groups, we used the Kruskal–Wallis analysis of variance as a non-parametric method. All statistical calculations and analyses were performed by using the IBM SPSS (PASW) Statistics (version 19.1). P value of less than 0.05 was considered significant.

**Table-I:** Characteristics of 286 cases of IE analyzed together and separately, in eight-year intervals.

	*1995 – 2010* *(n* ^1^ * = 286)*	*1995 – 2003* *(n = 162)*	*2004 – 2010* *(n = 124)*	*p value*
*Sex *				
Male	172 (60.1%)	90 (55.6%)	82 (66.1%)	0.363
Female	114 (39.9%)	72 (44.4%)	42 (33.9%)	0.02
Mean age (± SD) (years)		24.2 ± 11	39.4 ± 15	0.01
Mean duration of symptoms	13 ± 9	15.6 ± 8	10.5 ± 3	0.003
Clinical manifestations				0.152^2^
Fever	227 (79.4%)	127 (78.4%)	100 (80.6%)	
Loss of appetite	109 (38.1%)	63 (38.9%)	46 (37.1%)	
Sweating	87 (30.4%)	51 (31.5%)	36 (29.03%)	
weight loss	74 (25.8%)	41 (25.3%)	33 (26.6%)	
Arthralgia	46 (16.1%)	26 (16.05%)	20 (16.1%)	
Myalgia	35 (12.2%)	21 (12.9%)	14 (11.3%)	
Hepatoxegalty	48 (16.8%)	28 (17.3%)	20 (16.1%)	
Splenomegally	45 (15.7%)	23 (14.2%)	22 (17.7%)	
Splinter hemorrhage	25 (8.7%)	16 (9.9%)	9 (7.26%)	
Petechia	52 (18.2%)	30 (18.5%)	22 (17.7%)	
Clubbing	22 (7.7%)	12 (7.4%)	10 (8.06%)	
Janwages Lesion	5 (1.7%)	3 (1.85%)	2 (1.6%)	
Osler node	13 (4.5%)	8 (4.9%)	5 (4.03%)	
Roth’s spot	6 (2.1%)	3 (1.85%)	3 (2.42%)	

^1^n is the number of episodes.

^2^p value for overall group differences.

## Results

 From Jan. 1995 to Dec. 2010, a total number of 286 episodes of IE was reviewed from which 162 episodes were in the first eight-year period and 124 episodes were in the second one. Two hundred and four patients were diagnosed as definite IE and 82 patients as possible IE. There were 172 male patients with male to female ratio was 1.5:1. Mean age of all patients was 30.2 ± 16 years, age ranged from 3 to 81 years. There was a significant increase in the mean age of the patients in the second eight-year period of time compared to the first interval (24.2±11 vs 39.4±15 years old, p value = 0.01). The mean intervals between the initiation of the symptoms and the admission to the hospital in first and second eight-year intervals, were 15.6(±8) days and 10.5(±3) days, respectively (p value = 0.113). Collectively, the most frequent clinical presentation was fever (≥ 38° C) in 227(79.4%) of the patients followed by the loss of appetite in 109 (38.1%) of the patients. Table-I presents demographic and clinical characteristics of 286 cases of IE.

 The most frequently observed predisposing cardiac conditions were rheumatic heart disease among the patients in the first period of time (38.9%) and intravenous drug injection between the patients in succeeding eight years (41.9%). There was a significant increase in the prevalence of blood-born infections (HIV, HCV, and HBV) in second period of time compared with first eight-year period. Table-II shows all predisposing conditions of the patients.

***Microbiologic Findings: ***Of the total 286 episodes, blood cultures were negative in 68 (23.8%) of cases. The predominant causative microorganisms were Streptococcus viridans [58 (35.8%) patients] in the first eight-year period and Staphylococcus aureus [50 (40.7%) patients] in the second one. There was a significant increase in the prevalence of Staphylococcus aureus as causative agent during the second interval compared to the first one (40.7% vs 22.8%, p value = 0.01). Table-III presents the microbiologic and laboratory findings in detail.

***Echocardiographic Findings: ***Among the patients in the first eight-year period, 65 (40.1%) had mitral valve involvement, followed by aortic valve involvement in 59 (36.4%) patients. Aortic valve was the most frequently involved valve in the patients of the second eight-year period of time [49 (39.5%) patients], followed by mitral valve involvement in 32 (25.8%) patients. There was a significant increase in tricuspid valve involvement in this period compared with first eight-year period (22.6% vs 8.6%, p value = 0.02). Prevalence of multiple valve involvement in the first and the second intervals were 7.4% and 8.9%, respectively. The echocardiographic findings of the patients are summarized in Table-III.

**Table-II:** Predisposing conditions in 286 episodes of IE analyzed together and separately, in eight-year intervals.

	*1995 – 2010* *(n = 286)*	*1995 – 2003* *(n = 162)*	*2004 – 2010* *(n = 124)*	*p value*
*Cardiac condition*				
Congenital heart disease	25 (8.7%)	11 (6.8%)	14 (11.3%)	0.075
Rheumatic heart disease	83 (29%)	63 (38.9%)	20 (16.1%)	0.005
Acquired valvular disease (other than RHD)	51 (17.8%)	23 (14.2%)	28 (22.5%)	0.126
Prosthetic valve	40 (13.9%)	26 (16%)	14 (11.3%)	0.221
Mitral valve prolapse	71 (24.8%)	32 (19.8%)	39 (31.4%)	0.091
No underlying cardiac condition	16 (5.6%)	7 (4.3%)	9 (7.3%)	0.321
Intravenous drug injection	78 (27.3%)	26 (16.05%)	52 (41.9%)	0.01
Recent surgical procedures				
Dental procedure	95 (33.2%)	71 (43.8%)	24 (19.3%)	0.02
Gastrointestinal procedure	43 (15%)	23 (14.2%)	20 (16.1%)	0.631
Genitourological procedure	43 (15%)	22 (13.6%)	21 (16.9%)	0.452
Others	29 (10.1%)	20 (12.3%)	9 (7.26%)	0.065
*Comorbidities *				
Diabetes mellitus	22 (7.7%)	13 (8.02%)	9 (7.26%)	0.09
Malignancier	26 (9.1%)	15 (9.26%)	11 (8.9%)	0.114
Hepatitis B	44 (15.4%)	13 (8.02%)	31 (25%)	0.01
Hepatitis C	31 (10.8%)	9 (5.6%)	22 (17.7%)	0.01
HIV infects	11 (3.8%)	2 (1.2%)	9 (7.26%)	0.001
Collagen vascular disease	9 (3.1%)	6 (3.7%)	3 (2.42%)	0.067
None	143 (%)	104 (64.2%)	39 (7.26%)	0.02

**Table-III:** The microbiologic, laboratory and the echocardiographic findings in 286 episodes of IE analyzed together and separately, in eight-year intervals.

	*1995 – 2010 (n = 286)*	*1995 – 2003 (n = 162)*	*2004 – 2010 (n = 124)*	*p value*
*Microbiologic Findings*				
Staphylococcus averus	87 (30.4%)	37 (22.8%)	50 (40.7%)	0.01
Viridans streptococci	88 (30.8%)	58 (35.8%)	30 (23.8%)	0.07
Coagulase negative staphylococci	29 (10.1%)	15 (9.26%)	14 (11.3%)	0.322
Enterococcus Fecalis	8 (2.8%)	5 (3.09%)	3 (2.42%)	0.163
Other	10 (3.5%)	6 (3.7%)	4 (3.2%)	0.331
Negative	68 (23.8%)	42 (25.9%)	26 (20.97%)	0.336
*Laboratory Findings *				
Anemia	97 (33.9%)	50 (30.9%)	47 (37.9%)	0.632
Positive C-Reactive Protein	159 (55.6%)	86 (53.09%)	73 (58.9%)	0.412
Microscopic Hematuria	93 (32.5%)	50 (30.9%)	43 (34.7%)	0.537
Proteinvria > (1gr /day)	103 (36%)	42 (25.9%)	61 (49.2%)	0.04
Creatinine > 1.5mgr/dl	89 (31.1%)	37 (22.8%)	52 (41.9%)	0.01
*Echocardiographic Findings*				
*Affected Valves*				
Aortic	108 (37.8%)	59 (36.4%)	49 (39.5%)	0.944
Mitral	97 (33.9%)	65 (40.1%)	32 (25.8%)	0.071
Tricuspid	42 (14.7%)	14 (8.6%)	28 (22.6%)	0.02
Prosthetic	40 (13.9%)	26 (16%)	14 (11.3%)	0.221
More than one native valve	22 (7.7%)	12 (7.4%)	11 (8.9%)	0.672

**Table-IV:** Comparison of extra cardiac complications and mortality between the patients with vegetation size (VS) ≥1 cm with those with VS <1 cm.

	*VS < 1cm* *(n = 190)*	*VS ≥ 1cm* *(n = 96)*	*p value*
Embolic brain infarction	8 (4.2%)	17 (17.7%)	0.01
Intracranial hemorrhage	7 (3.7%)	19 (19.8%)	0.001
Transient ischemic attack	15 (7.9%)	36 (37.5%)	0.005
Meningitis	6 (3.2%)	19 (19.8%)	0.003
Splenic abcess	1 (0.53%)	5 (5.2%)	<0.001
Mortality	31 (16.3%)	33 (34.4%)	0.003

 It is worth mentioning that overall, the mean maximal dimension of the vegetation was 1.01 ± 0.2cm. In separate analysis, it was found that the mean maximal dimension of the vegetation was significantly higher in Jan.2003-Dec.2010 period than the one in Jan.1995-Dec.2002 interval (0.89±0.4cm vs 1.31±0.3cm, p value = 0.01). Also, the mean size of the vegetation was noticeably higher among IDUs than non-IDUs (1.53±0.1cm vs 0.76±0.2cm, p value < 0.001).

***Outcome: ***Sixty four patients (22.3%) died from which 42 patients belonged to the first eight-year period of time (mortality rate = 25.9%) and 22 ones were included in the second eight-year period (mortality rate = 17.7%). Thirty two deaths happened during hospital admission, 18 ones took place between 1 to 3 months and 14 ones occurred during three months to one year after the admission. There was no significant difference regarding the mortality rate between the conservatively treated and surgically treated patients (20.7% vs 22.9%, p value = 0.07), but compared to conservative treatment, more patients died with surgical treatment within one month of admission (54.3% vs 32.1%, p value = 0.041). Mortality rate was noticeably higher among patients with vegetation size ≥ 1cm compared to those with vegetation size < 1cm (34.4% vs 16.3%, p value = 0.003). Extra cardiac complications, were also significantly increased when the vegetation size was larger than 1cm. Table-IV compares mortality rate and extra cardiac complications in patients with vegetation size ≥1 cm with those with vegetation size <1 cm.

## Discussion

 Characteristics of infective endocarditis are changing all over the world. We reviewed 286 episodes of infective endocarditis over 16 years to evaluate the possible changes in the characteristics of IE in Iran including the changes of demographic, microbiologic, and echocardiogaphic characteristics, and outcome of the disease.

***Changes in demographic characteristics: ***The mean age of the patients with IE increased during last 16 years. Also, the comparison of two consecutive eight-year intervals of our study showed a significant increase in the mean age of the patients in the second period. Most of previous studies in developing countries reported RHD as the predisposing factor for IE in a high proportion of cases.^14^^-^^17^ Some of them reported it in as high as 53% of patients.^14^ In this study, the overall prevalence of RHD as the predisposing heart condition was 26.6%. Also, a noticeable decrease was observed in the IE episodes predisposed by RHD during the second eight-year period compared to the first one and the previous studies. Putting these facts together and considering the higher mean age of the patients in the second eight-year period, we can propose that the decrease in the prevalence of RHD caused by the improvement in the diagnosing and treatment strategies of Streptococcal pharyngitis could be an important explanation for the increase in the mean age of the patients. 

 Consistent with previous studies which evaluated the characteristics of IE in Iran during the last decade,^18^ this study leads us to conclude that significant increase in the IE cases was associated with intravenous drug injection during the second eight-year period compared to the first one. This is also compatible with the changes of IE profile in most of other courtiers particularly developed ones.^19^^,^^20^ On the other hand, compared to the previous studies in developed countries,^8^^,^^19^ we didn’t observe a significant increase in the IE episodes related to hemodialysis. This could be related to the relatively younger population of the IE patients among developing countries compared to developed ones.

***Changes in microbiologic characteristics: ***We observed an increasing trend for IE episodes caused by Staphylococcus aureus. Many previous studies from both developed and developing countries reported the same changing pattern^16^^,^^19^^,^^20^ This could be attributed to the increase in the population of IE patients with the history of intravenous drug injection.

***Changes in echocardiographic characteristics: ***Consistent with most of the previous studies reported from Iran and other developing countries,^15^^-^^17^ mitral valve was the overall most frequently involved cardiac valve in our study; but, when analyzed separately, tricuspid valve was involved more than the other valves during the second eight-year period of our study. This significant increase in the cases with tricuspid valve involvement was again because of the increase in the population of IDUs with IE in whom the right side of the heart and particularly tricuspid valve frequently got involved.

***Changes in outcome: ***Compared to previous studies in Iran,^14^^,^^18^ the mortality rate increased in our study. In separate analysis, the mortality rate was higher in second eight-year period than the first one. This increasing pattern in the mortality of IE is also reported by other studies from both developing and developed countries.^2^^,^^3^^,^^8^^,^^19^

 Some of the studies considered the specific characteristics of the disease as predictors of mortality. Cabell et al^8^ considered Staphylococcus aureus infection as an independent predictor of higher mortality whereas Thuny et al^21^ who reported vegetation size as a strong predictor of new embolic events and mortality. In our study, vegetation size was the most important prognostic predictor. We found that mortality and extra cardiac complications were significantly higher among the patients with vegetation size ≥ 1 cm. 

 All this shows that the characteristics of IE are changing all over the world but there are some differences between developed and developing countries. These changes seem to have contributed to the changes in our medical practice in post antibiotic era and the emergence of intravenous drug injection-associated episodes of IE. Furthermore, these changing characteristics have considerably influenced the outcome of the disease. Among these changing characteristics, vegetation size seems to have the greatest impact on the mortality and it could be considered as one of the outcome predictors in patients with infective endocarditis.
